# Establishing in vivo and ex vivo chick embryo models to investigate fetal tendon healing

**DOI:** 10.1038/s41598-023-35408-w

**Published:** 2023-06-13

**Authors:** Phong K. Nguyen, Christoph Hart, Kaitlyn Hall, Iverson Holt, Catherine K. Kuo

**Affiliations:** 1 Department of Biomedical Engineering, University of Rochester, Rochester, NY USA; 2 Center for Musculoskeletal Research, University of Rochester Medical Center, Rochester, NY USA; 3 Fischell Department of Bioengineering, University of Maryland, 4108 A. James Clark Hall, 8278 Paint Branch Drive, College Park, MD 20742 USA; 4 Department of Orthopaedics, University of Rochester Medical Center, Rochester, NY USA; 5 Department of Orthopaedics, University of Maryland School of Medicine, Baltimore, MD USA

**Keywords:** Biomedical engineering, Model vertebrates

## Abstract

Injured adult tendons heal fibrotically and possess high re-injury rates, whereas fetal tendons appear to heal scarlessly. However, knowledge of fetal tendon wound healing is limited due in part to the need for an accessible animal model. Here, we developed and characterized an in vivo and ex vivo chick embryo tendon model to study fetal tendon healing. In both models, injury sites filled rapidly with cells and extracellular matrix during healing, with wound closure occurring faster in vivo. Tendons injured at an earlier embryonic stage improved mechanical properties to levels similar to non-injured controls, whereas tendons injured at a later embryonic stage did not. Expression levels of tendon phenotype markers, collagens, collagen crosslinking regulators, matrix metalloproteinases, and pro-inflammatory mediators exhibited embryonic stage-dependent trends during healing. Apoptosis occurred during healing, but ex vivo tendons exhibited higher levels of apoptosis than tendons in vivo. Future studies will use these in vivo and ex vivo chick embryo tendon injury models to elucidate mechanisms of stage-specific fetal tendon healing to inform the development of therapeutic approaches to regeneratively heal adult tendons.

## Introduction

Tendons play a critical role in enabling body movements by transmitting forces from muscles to bones. Unfortunately, tendon injuries are highly prevalent and lead to scar tissue with inferior mechanical properties and high re-injury rates^1–3^. An estimated 65 million physician visits involve tendon and ligament injuries each year in the United States^4^. These include Achilles (calcaneal) tendon injuries, which are increasing in frequency^5^. High rates of injuries and poor healing ability have led to a critical need for approaches to regeneratively heal adult tendons.

In contrast to adult tendons, injured fetal sheep tendons appear to heal scarlessly^6,7^. Injured tendons of 80–85 gestational day sheep fetuses recovered normal extracellular matrix (ECM) organization after 7 days of healing, whereas adult tendons healed as scar tissue with disorganized ECM^6^. Injured adult tendons also possessed more immune cell infiltrates and elevated expression levels of inflammatory mediators compared to injured fetal tendons^7^. When injured fetal and adult sheep tendons were transplanted subcutaneously into adult severe combined immunodeficient (SCID) mice, which mount a compromised inflammatory response, fetal tendons still healed scarlessly whereas adult tendons healed with scar^7^. Based on these findings, the authors suggested scarless healing capacity is intrinsic to fetal tendons and not reliant upon the immune system, but the underlying mechanisms of fetal tendon scarless healing are still unknown. Uncovering mechanisms of fetal tendon scarless healing could inform the development of therapeutics to heal injured adult tendons regeneratively. To pursue such studies, a more accessible and economical animal model is needed.

We propose to use the chick embryo to study fetal tendon healing. The chick embryo is frequently used to study limb development because the process shares significant overlap with mammal^8–16^. However, unlike mammals, the chick embryo is attractive because it develops independently within an eggshell and its limb tendons can be accessed directly during development without the complications of in utero surgery^17^. Additionally, chick embryos are more economical than the sheep and many other models. We and others have used the chick embryo to elucidate key aspects of tendon development including collagen fibrillogenesis, collagen crosslinking, ECM contributions, and mechanical property elaboration^8–26^. Finally, the chick embryo develops in only 21 days. Taken together, the chick embryo is an attractive option to study mechanisms of fetal tendon healing.

Adult tendon healing includes inflammatory, proliferative, and remodeling phases (reviewed in^27,28^). It is unknown if these events also occur in fetal tendon healing. During the inflammatory phase of adult tendon healing, inflammatory cells release cytokines, leading to proliferation of tendon cells and recruitment of macrophages that produce additional pro-inflammatory mediators^29,30^. In adult tendons, interleukin (IL)-1β is one of the most significantly upregulated pro-inflammatory cytokines immediately following injury^31^. IL-1β binds IL-1 receptor 1 (IL-1R1) to transduce IL-1β signaling intracellularly^32^. IL-1β appears to regulate tendon phenotype as well as ECM elaboration during healing, as IL-1β treatment significantly downregulated *scleraxis*, *tenomodulin*, and *collagen types I* and *III,* and upregulated *mohawk* and *matrix metalloproteinase (MMP)13* expression in injured adult mouse Achilles tendon cells^33^. While collagen type I is the dominant fibrillar collagen type in healthy adult tendon, an abnormally low collagen type I-to-III ratio is associated with adult tendons after healing^34–36^. ECM remodeling during tendon healing appears to involve MMP3, MMP9, and MMP13 based on their expression patterns and effects of their inhibition on adult tendon healing^31,37–41^. It would be interesting to examine pro-inflammatory molecules, collagens, and MMPs in embryonic tendon healing.

Lysyl oxidase (LOX) is present during adult tendon healing, though its role is unknown^42^. Interestingly, LOX is hypothesized to play a role in fetal wound healing of other tissues. For example, LOX levels are higher in fetal mouse skin when wounded at embryonic day (E) 19 which heals with scar, compared to E16 which heals scarlessly^43,44^. Based on these results, LOX was hypothesized to play a role in stage-dependent healing of fetal skin, although perturbation studies were not performed to confirm this hypothesis. We previously discovered LOX plays a critical role in embryonic tendon development by regulating collagen crosslinking to control tendon mechanical properties^14,15^. Characterizations showed tendon elastic modulus correlates highly with LOX-mediated collagen crosslink density (r^2^ = 0.8, *p* < 0.0001) and LOX activity levels (r^2^ = 0.97, *p* = 0.016) during embryonic development^15,21^. Furthermore, inhibition of LOX activity by β-aminopropionitrile (BAPN) significantly decreased crosslink density and tendon elastic modulus without affecting collagen content or organization^14,15,21^. Given that LOX is a critical regulator of mechanical properties during tendon development, LOX and its regulators (BMP-1, fibronectin, periostin) should be investigated in embryonic tendon healing.

Expression of tendon phenotype markers, including scleraxis and tenomodulin, changes significantly after injury in adult tendons^31,45,46^. Knockdown of these markers or depletion of cells expressing these markers has led to altered adult tendon healing outcomes^35,47^. Specifically, depletion of scleraxis-expressing cells enhanced stiffness and maximum loads of injured adult mouse flexor tendons^35^. On the other hand, knockout of tenomodulin impaired adult mouse Achilles tendon healing and caused excessive fibrovascular scarring and heterotopic ossification^47^. Based on their roles in adult tendon healing, the roles of tendon phenotype markers in embryonic tendon healing should be explored.

Several studies with adult tendons have reported the presence of apoptotic cells during healing^48,49^, although the role of apoptosis in adult tendon healing is currently unknown. While not yet examined in embryonic tendon healing, apoptosis has been implicated in scarless healing of fetal skin healing based on higher expression levels of apoptosis markers in scarlessly healing E15 fetal mouse skin as compared to scarred healing E18 fetal skin^50^. It would be interesting to examine apoptosis in embryonic tendon healing after injury.

Here, we developed and characterized in vivo and ex vivo chick embryo models to study embryonic tendon healing. While the in vivo model provides a physiological environment, an ex vivo model enables greater control over the culture environment without the myriad of unknown and uncontrollable factors that are present in vivo. Tendons of Hamburger-Hamilton^51^ (HH) 40 and HH43 (incubation days 14 and 17) chick embryos were injured, and healing responses were assessed using histological staining and image analyses, gene expression analyses, and mechanical testing. We chose these stages because collagen content and LOX-mediated crosslink density increase by factors of 2 and 5, respectively, from HH40 to HH43^14,15^, and because HH43 is the first timepoint after HH40 when tensile modulus and peak stress become statistically different from HH40^23^. We hypothesized that embryonic tendons would heal rapidly after injury and that healing responses would be dependent on developmental stage. Our data establish the feasibility of chick embryo in vivo and ex vivo tendon injury models and reveal new insights into embryonic tendon healing. Future studies will use these models to identify critical regulators of embryonic tendon healing to inform the development of therapeutics for regenerative adult tendon healing.

## Methods

### Animals

All animal experiments were performed in accordance with relevant guidelines and regulations and received approval from the University of Maryland Institutional Animal Care and Use Committee (IACUC). All methods are reported in accordance with ARRIVE guidelines. HH40 and HH43 fertilized White Leghorn chicken eggs (University of Maryland Department of Animal and Avian Sciences) were cultured in humidified rocking incubators at 37.5 °C. At specified timepoints, chick embryos were sacrificed by decapitation, staged based on anatomical features, and used for ex vivo cultures and assays. 48 h and 120 h timepoints were selected based on when wound closure was evident in each model.

### In ovo injury

**Figure 1:**
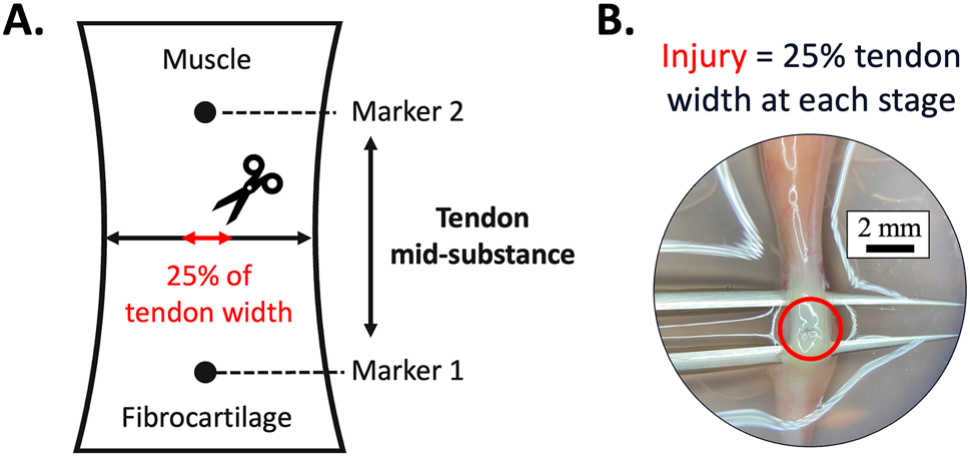
Injury was created by transecting 25% of tendon width at the midpoint of the calcaneal tendon mid-substance. (**A**) Markers 1 and 2 were placed to label the length of the tendon mid-substance, excluding adjacent muscle and fibrocartilage, using an established protocol^23^. The midpoint of the tendon mid-substance was identified using calipers that were set to one half the length of the mid-substance, and then to one half the length of the mid-substance width. At this midpoint, a pair of micro scissors was used to transect 25% of tendon width. (**B**) Photo of an injury (within red circle) in the calcaneal tendon of an HH43 embryo (India ink not included for better viewing of the tendon).

At HH40 and HH43, a 3 cm-diameter window was created over the air cell. A high temperature cautery (Bovie) created a 0.5 cm-diameter opening in the chorioallantoic membrane while cauterizing severed blood vessels. A V-hook lifted the limb through the opening in the membrane, and a custom-made brace held the ankle at a 90° angle above the window. Our established “marking protocol”^23^ identified and used India ink to mark the length of the calcaneal tendon mid-substance based on anatomical features (Fig. 1). We identified the midpoint of the tendon mid-substance using calipers set to one half the length and then to one half the width of the mid-substance. At the midpoint, micro-scissors dipped in India ink created an injury site spanning 25% of the tendon width (Fig. 1). A custom metal tool inserted between the tibia and tendon protected the bone during the transection. The limb with the tendon injury was returned below the membrane. The intact contralateral tendon was the non-injured control. We covered the window with tape and incubated the egg in a humidified incubator at 37.5 °C. After 48 h, embryos were sacrificed and calcaneal tendons harvested for the assays.

### Ex vivo injury

HH40 and HH43 limbs were harvested by cutting halfway along the tibia length and removing the digits of the foot. The skin was removed but muscles and bones were kept intact to maintain the resting tension on the calcaneal tendon. Limbs were placed in sterile saline and the ankle held at a 90° angle to demarcate the calcaneal tendon mid-substance using the marking protocol^23^ and create an injury (described above, Fig. 1). The contralateral limb was the non-injured control. Limbs were cultured in Dulbecco’s modified Eagle’s medium (Life Technologies) with 10% fetal bovine serum (Atlanta Biologicals) and 1% antimycotic-antibiotic (Gibco). Medium was changed every 12 h. Calcaneal tendons were harvested for RT-PCR at 12 h; histology and imaging at 48 h and 120 h; and tensile mechanical testing at 0 h and 120 h.

### Histology, imaging, and quantification

Tendons were cryopreserved and cryosectioned at 20 μm thickness, as previously described^14,24^. Injury sites were identified by India ink and the tissue depth of these sections recorded. Contralateral non-injured control sections at the same tissue depth as in the injured samples were selected for staining alongside injured samples. Hematoxylin and eosin (H&E) staining were used to quantify cell density, and picrosirius red (PSR) staining to quantify collagen properties, as previously described^13,14,22,24^. Stained sections were imaged under brightfield and polarized light microscopy (20X objective, ZEISS Axio Scan) and shown with the wound site only (without adjacent uninjured tissue). Briefly, cells were counted in H&E-stained sections by three reviewers in a blinded fashion using the Cell Counter plug-in in ImageJ (NIH)^22,24^. To quantify collagen content in PSR-stained sections, brightfield images were converted to RGB stacks using ImageJ (NIH) and the total signal intensity in the red channel was measured^22,24^. To quantify collagen density, the percentage of PSR-positive staining areas per image was measured after applying the same thresholding to every image to distinguish PSR-positive from PSR-negative staining areas. To quantify collagen alignment, brightfield images were converted into 8-bit format, and fiber orientation was assessed using the Directionality plugin in FIJI (NIH). We quantified collagen maturity based on percentage of green, yellow, and red pixels as immature, intermediate, and mature fibers, as previously described^24^. Briefly, polarized images were converted into 8-bit format. A custom MATLAB code classified a pixel as red if the intensity ratio of red to green signal (I_r_/I_g_) ≥ 1.8, yellow if 1.1 < I_r_/I_g_ < 1.8, and green if I_r_/I_g_ ≤ 1.1. We stained and quantified apoptotic cells using the In Situ Cell Death Detection Kit (Roche) and all cell nuclei with Hoechst 33,342 (Thermo Fisher), as previously described^24^. Red-fluorescing TUNEL-positive cells and blue-fluorescing Hoechst 33,342-positive nuclei (pseudo-colored green in images) were imaged on a FLUOVIEW FV3000 laser scanning confocal system (Olympus) and counted using the Analyze Particle function in ImageJ (NIH). Apoptotic cell percentage was calculated by dividing TUNEL-positive cell number by Hoechst 33,342-positive cell number.

### Second Harmonic Generation (SHG) imaging and quantification

Fibrillar collagen was visualized in sections by forward SHG (800 nm excitation, 400 ± 10 nm emission; TCS SP8 MP multiphoton microscope, Leica) with a 100X oil immersion objective and analyzed, as previously described^14–16,21,24^. Briefly, collagen content was quantified as the total SHG signal intensity per image (Image J). Collagen density was quantified as the percentage of SHG signal-positive area per image after applying the same thresholding to every image to distinguish SHG signal-positive from signal-negative areas. Collagen alignment was quantified using the Directionality plugin in FIJI (NIH) that generated an average fiber dispersion per image.

### Mechanical testing

Tendons were tensile tested as previously described^23,24^ by stretching uniaxially at 1% strain/s until failure (5542 Advanced Material Testing System, Instron). Elastic modulus was calculated from the slope of the linear region of the stress–strain curve. Peak stress was the maximum load normalized to tendon cross-sectional area. Peak strain was determined as the strain value at peak stress.

### Reverse‐transcription polymerase chain reaction (RT‐PCR)

Tendons were homogenized in TRIzol LS (Life Technologies), total RNA isolated and reverse-transcribed using the Superscript III First Strand Synthesis kit (Invitrogen), and PCR performed with PlatinumTM Taq DNA Polymerase High Fidelity (Invitrogen) using a Mastercycler® (Eppendorf). Gallus gallus-specific primers (newly and previously designed^21,25,52^) were used to characterize gene expression levels (Table 1).

**Table 1:** Forward and reverse primer sequences.

Gene	Accession #	Forward sequence	Reverse sequence
*18 s ribosomal RNA gene (18S)*^21,25,52^	AF173612.1	CGGGGCCATGATTAAGAGGG	CTTTAGTTCGTCTTGCGCCG
*Bone morphogenic protein 1 (BMP1)*	U75331.1	GTTCTGCGGCTACGAGAAAC	AACATTCGTCCACCTCCTTG
*Collagen type I alpha 2(I) chain (COL1A2)*^52^	NM_001079714.1	CCAGGACAACCTGGTGCTCGC	CAGCGCTGCCATCACTCCCA
*Collagen type III alpha 1 chain (COL3A1)*^52^	NM_205380.2	TTCAGGAGCAAGGGGTCCACC	AGGGAAGCTACGCCACCACCA
*Fibronectin* (FN1)	NM_001198712.1	CGTTCGTCTCACTGGCTACA	GGTCCTCTGGATGGGATTCT
*Interleukin 1 beta (IL-1β)*	NM_204524.2	CCAGAAAGTGAGGCTCAACA	GTAGCCCTTGATGCCCAGT
*Interleukin 1 receptor type 1 (IL-1R1)*	NM_205485.1	TGATTCTCAAGAATTTACATCATACAT	CTTCTCCTGCTAAATCATTCCTC
*Lysyl oxidase (LOX)*^21,52^	NM_205481.2	TCGGGCGGATGTTAGAGACT	AGCTGGCGTCTAACAAGTCA
*Matrix metalloproteinase 3 (MMP3)*	XM_417175.2	ATCAGGCTCTACAGTGGTG	ATGGGATACATCAAGGCAC
*Matrix metalloproteinase 9 (MMP9)*	NM_204667.1	ATGAACTACTCCCCCGACCTG	AGTCCAGAACTCATCATCATCG
*Matrix metalloproteinase 13 (MMP13)*	NM_001293090.1	TTTGGGCTATGAATGGCTAT	TAGTATGCAGGATGCGGACA
*Mohawk homeobox transcript variant X1 (MKX)*	XM_015282064.3	AGGATTACGTGTCACCTCCC	TCTGTTAGCTGCGCTTTCAC
*Periostin (POSTN)*	NM_001030541	GTGCTGTCCTGGCTACATGA	TGTGGTGGTGGAGAGCATTA
*Scleraxis (SCX)*^25,52^	NM_204253.1	CGCGACAGGAAGACGGCGAT	CTGGCAGCGGGGTGAAGACG
*Tenascin-C (TNC)*	NM_205456.4	GCGGCTACAACAGAGGCAG	CCCATCATCTGCAGTCCAGG
*Tenomodulin (TNMD)*^25,52^	NM_206985.2	CATGGTCTGGGTGCCTGGCG	TCCGGAGCTGCTATCGGGGT

### Statistical analyses

Power analysis was performed with power = 0.9 and significance level α = 0.05 using 1 sample, 2-sided equality, and 2-sample equivalence scenarios. Effect size was 1.1 for tensile testing and 1.8 for RT-PCR and image analysis data. Based on power analysis results, N = 5 tendons for tensile mechanical testing and N = 3 tendons for image analysis and RT-PCR were required per condition and embryonic stage. Each biological replicate (N) was from a different animal. Final data set was tested for and met both normality and homogeneity of variance criteria. Two-tailed Student t-test was used to analyze statistical differences between injured and non-injured tendons at the same timepoint and injured tendons at different timepoints. One-way ANOVA followed by Tukey’s test (for nuclei density comparisons) and two-way ANOVA followed by Sidak's test (for viability and mechanical property comparisons) were used to analyze statistical differences between injured and non-injured tendons at different timepoints. Statistical analyses were performed using Graphpad Prism v8 (CA, United States).

## Results

**Figure 2:**
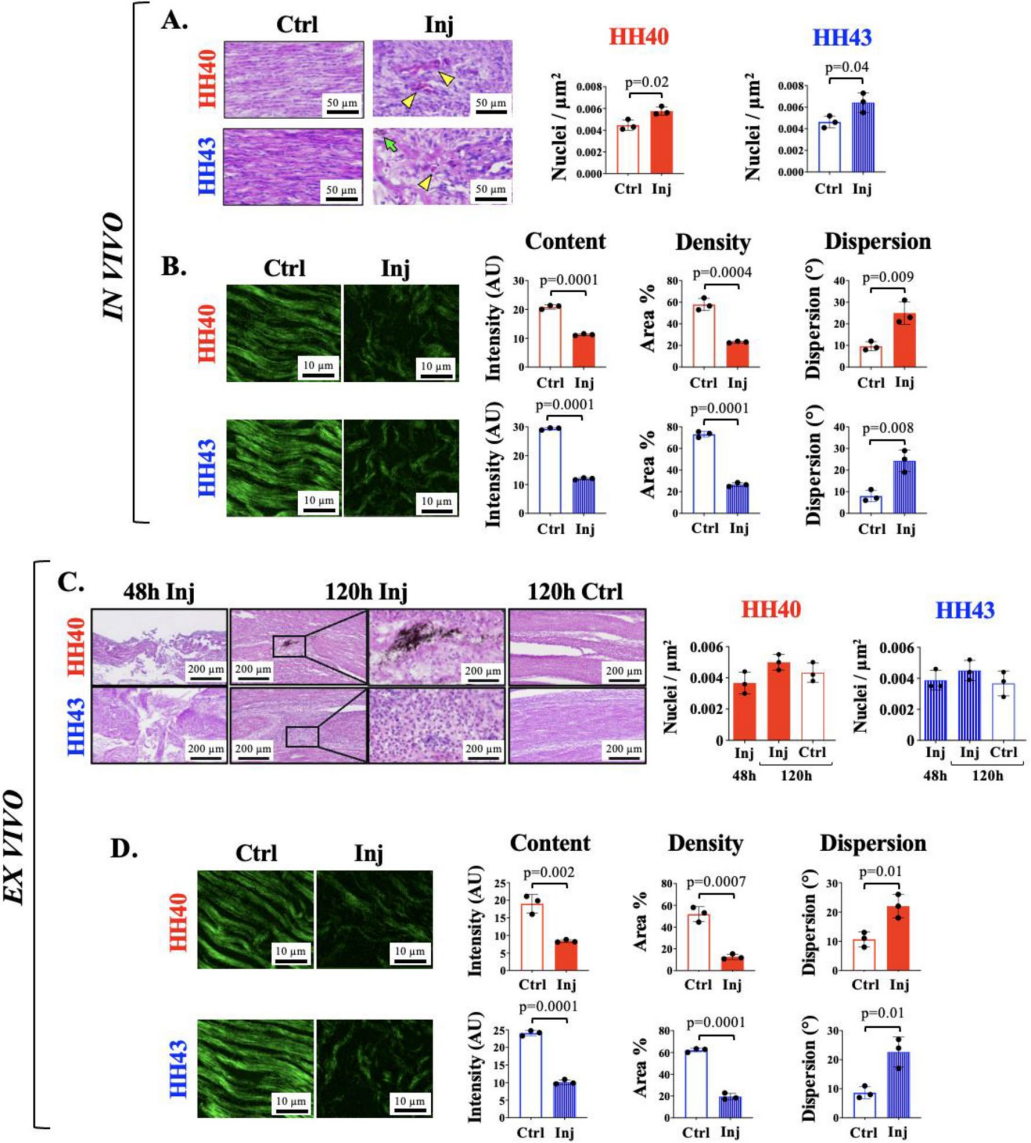
H&E staining and SHG imaging revealed cells and disorganized fibrillar collagen filled in HH40 and HH43 tendon wounds in vivo by 48 h (**A**,**B**) and ex vivo by 120 h (**C**,**D**)*.* (**A**) Representative H&E stains and image analyses show in vivo injured HH40 and HH43 tendon wounds filled by 48 h, and that wounds had higher cell density than non-injured controls at 48 h. Potential immune cells were also observed. Green arrows point to India Ink. Yellow arrowheads point to potential immune cells. (**B**) Representative SHG images and image analyses of in vivo injured HH40 and HH43 tendon wound sites at 48 h show lower fibrillar collagen content and density but higher dispersion than non-injured controls (*p* < 0.05). (**C**) Representative H&E stains and image analyses show ex vivo injured HH40 and HH43 tendons wounds filled by 120 h, and that wounds had similar cell densities between 48 and 120 h. At 120 h, cell densities were also similar between injured and non-injured tendons for both HH40 and HH43. (**D**) Representative SHG images and image analyses of ex vivo injured HH40 and HH43 tendon wound sites at 120 h show lower fibrillar collagen content and density but higher dispersion than non-injured controls (*p* < 0.05). Statistically significant differences between injured and non-injured tendons of the same stage at the same timepoint and between injured tendons of the same stage at different timepoints were determined by two-tailed Student t-test with *p* < 0.05. N = 3 per stage and condition.

H&E staining and SHG imaging showed wounds filled with cells and disorganized ECM by 48 h in vivo and 120 h ex vivo, with fibrillar collagen as a significant component of the ECM (Fig. 2). In vivo, injured HH40 and HH43 tendon cell densities were 129% and 139% of non-injured controls, respectively (Fig. 2A). Based on SHG imaging, fibrillar collagen content, density, and dispersion in in vivo injured HH40 tendons were 54%, 40%, and 259% of non-injured controls, respectively (Fig. 2B), and in HH43 injured tendons were 41%, 36%, and 304% of non-injured controls, respectively (Fig. 2B).

Ex vivo, injured tendon cell densities were similar between 48 and 120 h for both HH40 and HH43 (Fig. 2C). At 120 h, cell densities were not different between injured and non-injured tendons for both HH40 and HH43 (Fig. 2C). Based on SHG imaging, fibrillar collagen content, density, and dispersion in injured HH40 tendons were 44%, 24%, and 206% of non-injured controls, respectively, and in injured HH43 tendons were 42%, 31%, and 262% of non-injured controls, respectively (Fig. 2D).

**Figure 3:**
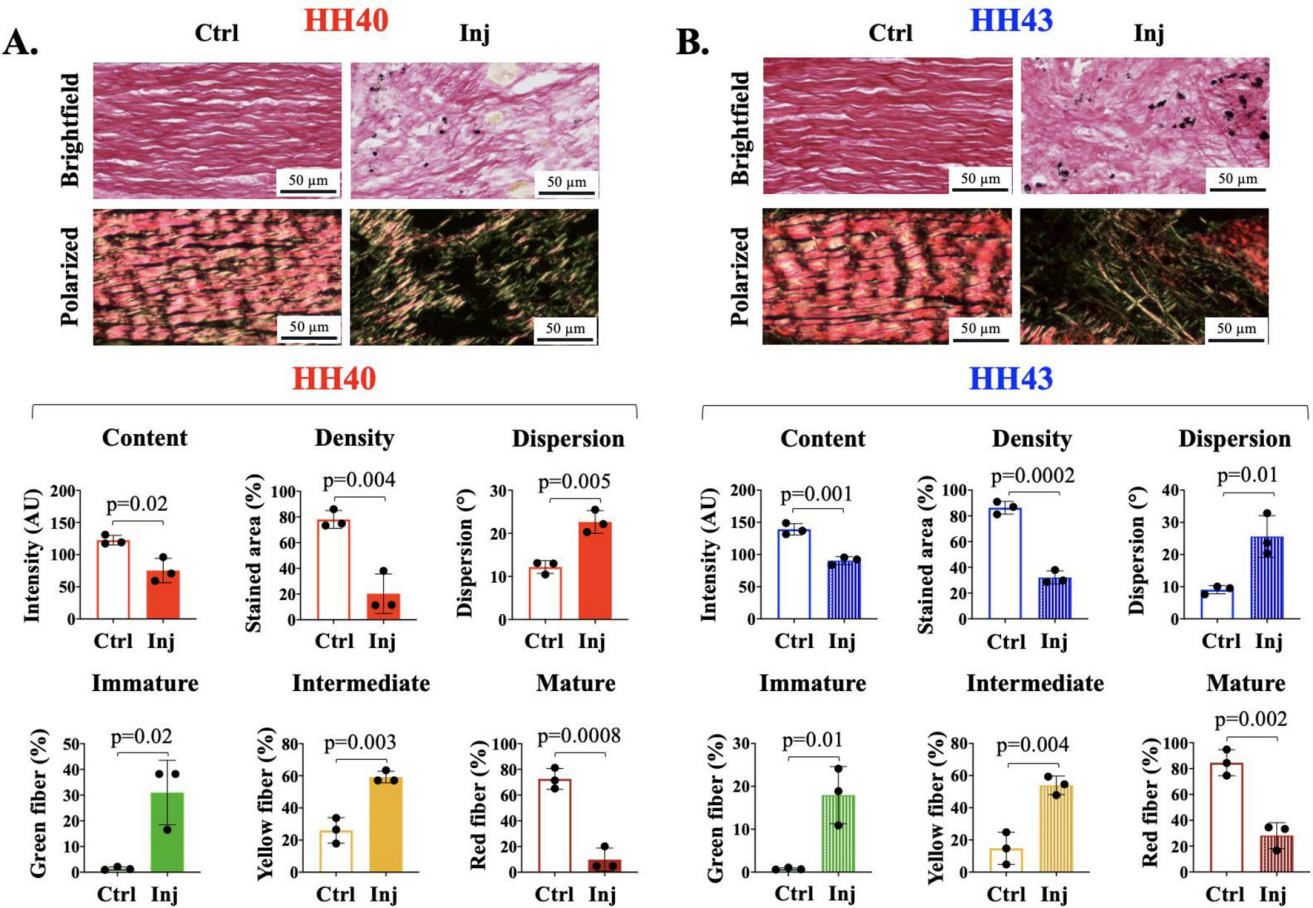
PSR staining and polarized microscopy revealed HH40 and HH43 tendon wounds in vivo possessed mostly immature and intermediate collagen fibers at 48 h. Representative PSR stains and image analyses for collagen content, density, dispersion, and fibril maturity of HH40 (**A**) and HH43 (**B**) tendons injured in vivo. Both HH40 (**A**) and HH43 tendons (**B**) possessed disorganized collagen at 48 h that was lower collagen content and density, but higher dispersion than non-injured controls (*p* < 0.05). Polarized microscopy and image analyses revealed injured HH40 (**A**) and HH43 tendons (**B**) possessed more immature and intermediate collagen fibers and fewer mature collagen fibers in comparison with non-injured controls (*p* < 0.05). Statistically significant differences between injured and non-injured tendons at each stage were determined by two-tailed Student t-test with *p* < 0.05. N = 3 per stage and condition.

We performed PSR staining of in vivo injured tendons to analyze collagen content and maturity at 48 h of healing (Fig. 3). Relatively high content of immature and intermediate collagen fibers suggested healing was ongoing in both HH40 and HH43 injured tendons. Specifically, collagen content, density, and dispersion in HH40 injured tendons were 61%, 26%, and 186% of non-injured controls, respectively (Fig. 3A), and in HH43 injured tendons were 65%, 37%, and 283% of non-injured controls, respectively (Fig. 3B), which agreed with SHG imaging analyses (Fig. 2). Polarized microscopy revealed immature, intermediate, and mature collagen fibers in injured HH40 tendons were 2,190%, 228%, and 14% of non-injured controls, respectively (Fig. 3A), and in injured HH43 tendons were 2,383%, 366%, and 33% of non-injured controls, respectively (Fig. 3B).

**Figure 4:**
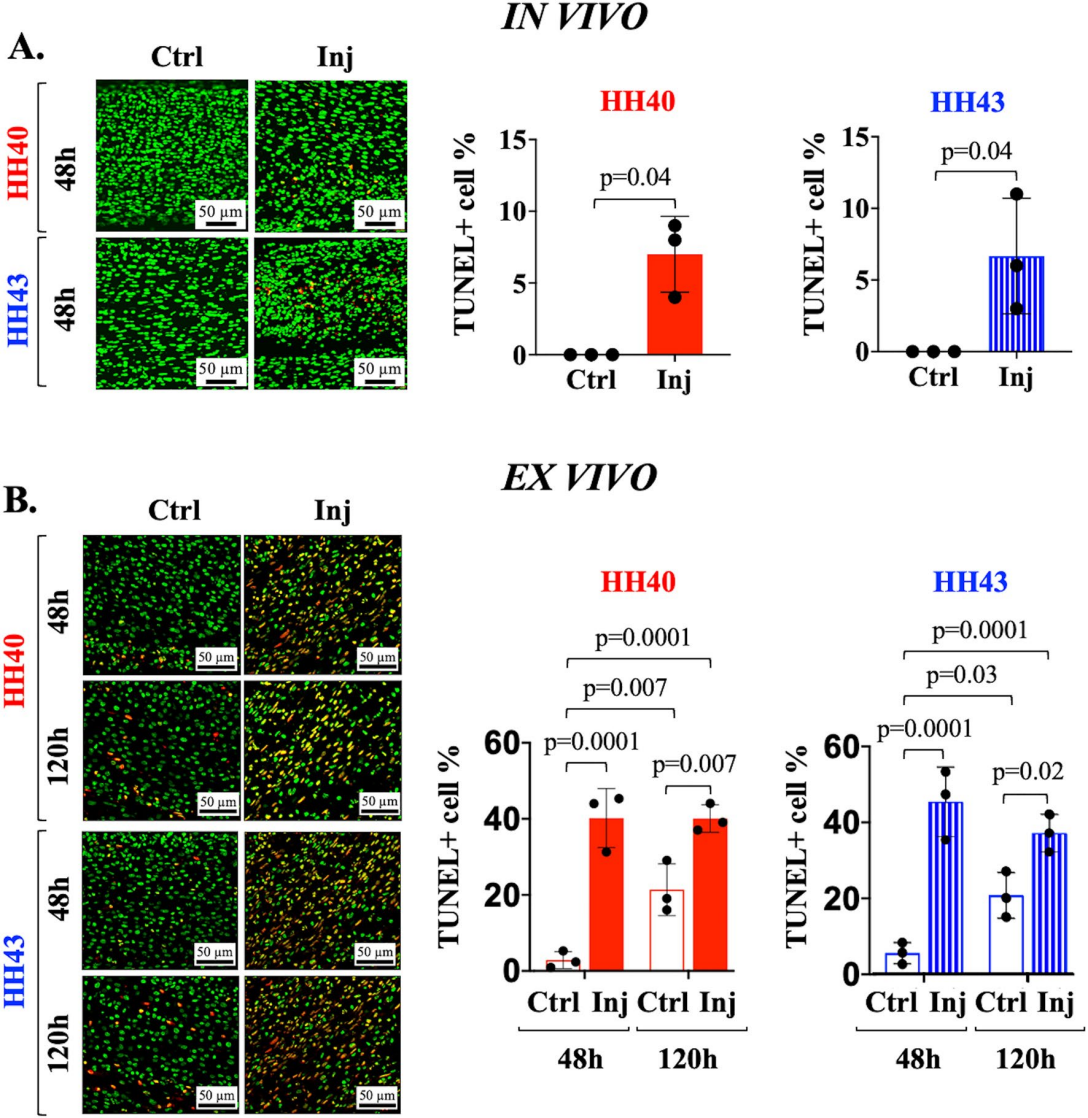
Apoptosis was higher in HH40 and HH43 tendon wounds than in contralateral non-injured tendons both in vivo and ex vivo. Representative TUNEL stains and image analyses shown for in vivo (**A**) and ex vivo (**B**) injured tendons. (**A**) In vivo injured HH40 and HH43 tendon wounds possessed apoptotic cells whereas non-injured controls had none at 48 h (*p* < 0.05). (**B**) Ex vivo injured HH40 and HH43 tendon wounds possessed higher TUNEL-positive cell percentages than non-injured controls at 48 h and 120 h (*p* < 0.05). In addition, TUNEL-positive cell percentages in non-injured HH40 and HH43 tendons increased from 48 to 120 h (*p* < 0.05). Statistically significant differences between injured and non-injured tendons at each stage in vivo were determined by two-tailed Student t-test with *p* < 0.05. Statistically significant differences between injured and non-injured tendons at each stage and between timepoints ex vivo were determined by 2-way ANOVA followed by Sidak's multiple comparisons test with *p* < 0.05. N = 3 per stage and condition.

TUNEL staining revealed differences in the in vivo and ex vivo models. HH40 and HH43 tendons injured in vivo both possessed 7 ± 2% TUNEL-positive cells compared to 0% in non-injured controls by 48 h (Fig. 4A). Ex vivo, injured HH40 tendons possessed 40 ± 6% and 40 ± 3% TUNEL-positive cells at 48 h and 120 h, respectively. HH40 non-injured controls possessed 3 ± 2% TUNEL-positive cells at 48 h and increased to 21 ± 6% TUNEL-positive cells by 120 h (Fig. 4B). HH43 tendons injured ex vivo possessed 45 ± 8% and 37 ± 4% TUNEL-positive cells at 48 h and 120 h, respectively. HH43 non-injured controls possessed 5 ± 2% TUNEL-positive cells at 48 h and increased to 21 ± 5% TUNEL-positive cells by 120 h (Fig. 4B).

**Figure 5:**
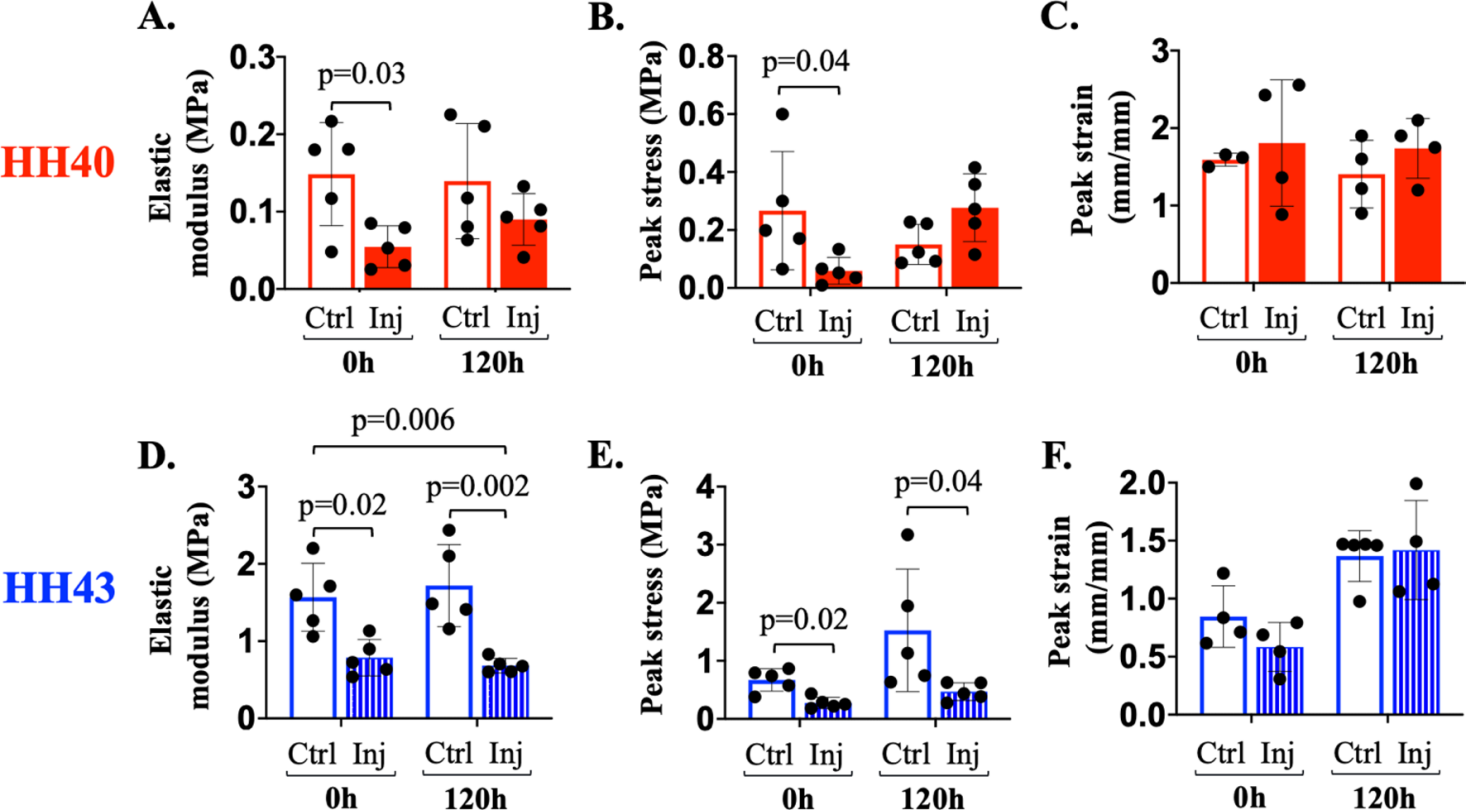
Tensile mechanical properties of ex vivo injured HH40 tendon improved to the same levels as non-injured controls by 120 h, whereas HH43 tendons did not. Injured HH40 tendon elastic modulus (**A**) and peak stress (**B**) were lower than those of non-injured controls at 0 h (*p* < 0.05), but increased to the same levels of non-injured controls by 120 h (*p* > 0.05). Injured HH40 tendon peak strains were similar to non-injured controls at 0 h and 120 h (*p* > 0.05) (**C**). Injured HH43 tendon elastic modulus (**D**) and peak stress (**E**) were lower than those of non-injured controls at both 0 h and 120 h (*p* < 0.05). Injured HH43 tendon peak strains were similar to non-injured controls at 0 h and 120 h (*p* > 0.05) (**F**). Statistically significant differences between injured and non-injured tendons at each stage and between timepoints were determined by 2-way ANOVA followed by Sidak's multiple comparisons test with *p < 0.05. N = 5 per stage and condition.

To analyze mechanical properties during healing, uniaxial tensile testing was performed with ex vivo tendons. Injury immediately compromised mechanical properties of both HH40 and HH43 tendons, but only HH40 injured tendons exhibited improvements during healing (Fig. 5). Specifically, HH40 injured tendon elastic modulus was 37% and peak stress was 24% of non-injured controls at 0 h, but by 120 h elastic modulus and peak stress were similar to non-injured controls (Fig. 5A,B), whereas peak strains appeared unaffected by injury or time (Fig. 5C). Injured HH43 tendon elastic modulus was 50% and peak stress was 41% of non-injured controls at 0 h (Fig. 5D,E). At 120 h, elastic modulus and peak stress of HH43 injured tendons still were 40% and 31% of non-injured controls, respectively (Fig. 5D,E), whereas peak strains appeared unaffected by injury or time (Fig. 5F).

**Figure 6:**
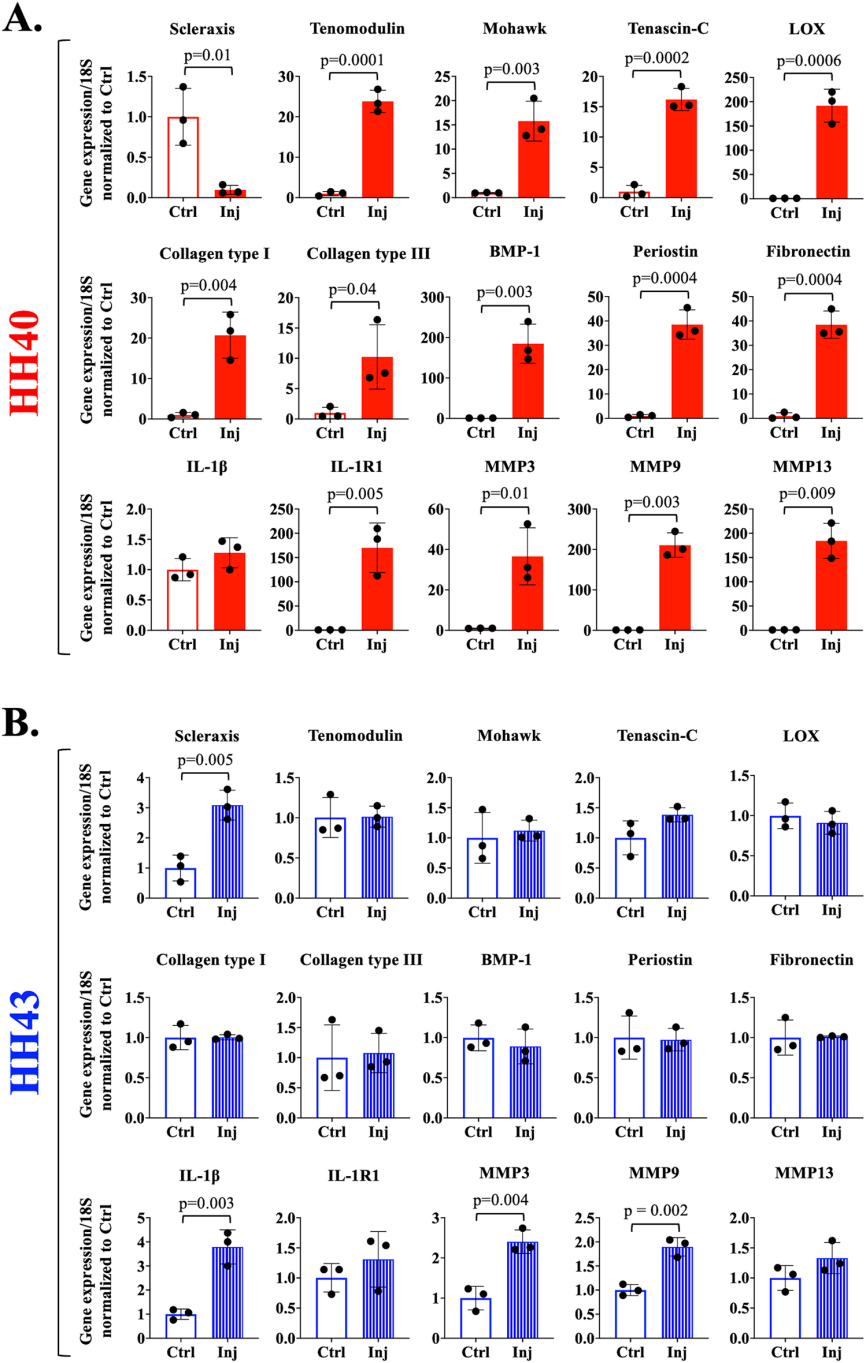
Gene expression levels were differentially regulated in HH40 and HH43 tendons after injury ex vivo. (**A**) In injured HH40 tendons, *scleraxis* expression levels were lower than in non-injured controls (*p* < 0.05). In contrast, *IL-1β* expression levels were similar between injured and non-injured HH40 tendons (*p* > 0.05), and expression levels of all other markers were higher than in non-injured controls (*p* < 0.05). (**B**) In injured HH43 tendons, *scleraxis, IL-1β, MMP3,* and *MMP9* expression levels were higher than in non-injured controls (*p* < 0.05). In contrast, expression levels of all other markers were similar between injured and non-injured HH43 tendons. Statistically significant differences between injured and non-injured tendons were determined by two-tailed Student t-test with *p* < 0.05. N = 3 per condition.

To further investigate differences in HH40 and HH43 tendon healing, we examined gene expression levels of a range of molecules. In HH40 tendons injured ex vivo, *scleraxis* expression was lower than in non-injured controls (Fig. 6A). In contrast, *tenomodulin, mohawk, tenascin-C, IL-R1, collagen types I and III, MMP3, MMP9, MMP13, LOX, BMP-1, fibronectin,* and *periostin* were higher than in non-injured tendons, and *IL-1β* levels were not affected (Fig. 6A). In HH43 tendons injured ex vivo, *scleraxis, IL-1β, MMP3,* and *MMP9* expression levels were higher than in non-injured controls, whereas expression levels of the other markers were not affected (Fig. 6B).

## Discussion

This study established in vivo and ex vivo chick embryo tendon injury models and used these models to discover insights into stage-specific events of embryonic tendon healing. We demonstrated the ability to create a partial thickness wound in the mid-substance of the calcaneal tendon at earlier and later stages of development and observed rapid wound closure both in vivo and ex vivo. While healing was more rapid in vivo than ex vivo, we did not observe scarless or regenerative healing at the timepoints examined. Whether chick embryonic tendons heal regeneratively will be examined in future studies. We developed the ex vivo model to study healing in isolation of unknown and uncontrollable factors that are present in vivo. Our data also suggest embryonic tendon responses to injury are stage-specific, which will be interesting to investigate further. Based on our findings, the in vivo and ex vivo chick embryo tendon injury models will be useful for future studies to elucidate key mechanisms and regulators of embryonic tendon healing.

Wounds filled with cells and ECM after injury in both HH40 and HH43 tendons by 48 h in vivo and 120 h ex vivo. Because the same initial wound-to-tendon width ratio was created in both models (Fig. 1), a difference in wound size was not the reason for different healing rates. Slower healing ex vivo could be due to reduced nutrient supply and waste exchange and other compromised factors. PSR staining and SHG imaging indicated collagen was a significant component of the new ECM in HH40 and HH43 wounds, although lower content and maturity and less organized in both in vivo and ex vivo injured tendons compared to non-injured controls (Figs. 2 and 3). It is possible the ECM will become more collagenous, dense, and organized with longer time to heal, as others have shown fetal sheep tendon can heal scarlessly. In particular, 80 gestation day sheep fetuses healed partially transected tendons in 7 days with recovery of normal ECM organization in utero^6^. Future studies should examine collagen elaboration over longer healing durations. It would also be interesting to examine how different wound sizes heal, as previous studies report fetal tendon scarless healing ability is dependent on wound size^53^.

Similar to adult tendon healing, embryonic tendons injured in vivo exhibited increases in cell density and apoptosis by 48 h of healing. In vivo injured HH40 and HH43 tendons possessed higher cell densities than non-injured controls (Fig. 2). Furthermore, apoptotic cells were observed in injured tendons only (Fig. 4). During adult tendon healing, resident tendon cells and immune cells increase in number and release factors that affect ECM synthesis^27,28,54^. Adult tendons also experience apoptosis during healing^48,49,55^. For example, TUNEL-positive cells were reported to be 1.6-fold higher in injured human Achilles tendons compared to healthy controls^48^. Fetal tendon healing studies have not examined apoptosis^6,7,53^, but a study in fetal skin reported higher levels of apoptosis markers caspase 7 and poly ADP-ribose polymerase in scarlessly healing E15 fetal mouse skin compared to scarred healing E18 skin^50^. Future studies should examine a potential role for apoptosis in stage-specific embryonic tendon healing at later timepoints.

More apoptotic cells in ex vivo injured tendons than non-injured controls suggests injury triggered apoptosis. In addition, ex vivo injured tendon viabilities were similar between HH40 and HH43 and remained constant over time despite different expression profiles of pro-inflammatory mediators (Figs. 4 and 6). Meanwhile, non-injured tendon cell viability declined slowly from 95 to ~ 79% from 48 to 120 h (Fig. 4). Our results differ from a report in which tendons of adult bone-tendon-muscle explants decreased rapidly to 0% viability by 120 h^56^. Future studies using the ex vivo model should consider viability over time.

Mechanical properties of ex vivo injured HH40 tendons became similar to those of non-injured controls by 120 h of healing, whereas those of HH43 tendons did not (Fig. 5). We would expect non-injured tendon mechanical properties to elaborate over 120 h if the tendons were developing normally, as occurs in vivo^23^. However, lack of increases in elastic modulus and peak stress in control tendons suggests compromised development ex vivo, which was also reflected by the increase in apoptosis over time (Fig. 4 and 5). Interestingly, injured HH40 tendon mechanical properties were statistically similar to control tendons at 120 h despite abnormally low collagen content and disorganized collagen, whereas injured HH43 tendon mechanical properties remained inferior to controls (Fig. 2 and 5). Normally, HH43 tendons have more developed ECM and mechanical properties than HH40 tendons^14,23^, thus after injury may need additional time to synthesize, remodel, and crosslink collagen to achieve similar properties as control tendons. Previously, we discovered that tendons with normal collagen content and organization will have inferior mechanical properties if LOX-mediated crosslink density is abnormally low^14,15^, and that rLOX treatment of tendons can increase LOX-mediated crosslink density to, in turn, enhance mechanical properties^24^. In vivo, active LOX enzyme is produced when bone morphogenetic protein (BMP)-1 cleaves proLOX^57–59^, a process that is enhanced via interactions between BMP-1 with fibronectin and periostin^58,60,61^. Here, *LOX*, *BMP-1*, *fibronectin*, and *periostin* levels increased in healing HH40 tendons relative to non-injured controls, but not in HH43 tendons (Fig. 6). Based on these results, we hypothesize that HH40 tendons increased LOX-mediated collagen crosslinking during healing to improve mechanical properties, despite abnormal collagen content and organization. Future studies should perturb LOX in vivo to test how LOX-mediated crosslinking of collagen regulates embryonic tendon mechanical properties during healing in a stage-specific manner.

HH40 tendon healing may have involved more active collagen synthesis and remodeling by MMPs than HH43 tendons. HH40 tendons upregulated *collagen types I and III, and MMP3*, *MMP9*, and *MMP13* during healing (Fig. 6a). In contrast, healing HH43 tendons only upregulated *MMP3* and *MMP9*, and to a lesser extent (Fig. 6b). Perhaps regulation of *collagen types I* and *III* contributed to the improvements in healing HH40 tendon mechanical properties. Collagen type III is present in embryonic tendons and may play a role in regulating collagen fibril diameter^9,13^. During healing, adult tendons possess a high collagen type III-to-I ratio^34–36^, but persistence of the high ratio after healing is associated with abnormal tendon function and health^9,34,62^. Future studies should examine collagen type III contributions to embryonic tendon healing. It is also possible that MMP remodeling of ECM played a role in HH40 tendon healing. MMPs have been implicated in adult tendon healing across tendon types^31,37–41^. For example, MMP inhibition by doxycycline compromised injured adult rat Achilles tendon mechanical properties^41^. Having previously detected active and pro-forms of MMPs in embryonic tendons^22,63^, it would be interesting to determine the roles of MMPs in embryonic calcaneal tendon healing.

Phenotype markers exhibited stage-dependent expression patterns during HH40 and HH43 tendon healing. *Scleraxis* decreased during HH40 tendon healing, while *tenomodulin* and other phenotype marker levels increased (Fig. 6a). In contrast, *scleraxis* levels increased during HH43 tendon healing while other phenotype markers were unaffected (Fig. 6b). Although not previously examined in embryonic healing, phenotype markers may play roles in postnatal tendon healing^31,35,45–47^. For example, injured adult mouse flexor tendons improved stiffness and maximum loads when scleraxis-expressing cells were depleted^35^. It is interesting that in our study, injured HH40 tendons downregulated *scleraxis* expression levels and improved mechanical properties to the same levels as non-injured controls (Fig. 5). In contrast, injured HH43 tendons, which upregulated *scleraxis* expression levels, did not improve mechanical properties (Fig. 5). Future studies should perturb scleraxis and other tendon markers to determine their roles in embryonic tendon healing.

IL-1β signaling may have influenced HH40 tendon healing outcomes. We examined *IL-1β* and *IL-1R1* as early markers of inflammation. IL-1β is one of the most upregulated and earliest detected pro-inflammatory cytokines after adult tendon injury^31^. IL-1β effects on embryonic tendon healing are unknown, but IL-1β-treated embryonic and postnatal tendon cells both upregulate *IL-1R1*^64^, which transduces IL-1 signaling. Injured adult mouse Achilles tendon cells respond to IL-1β by downregulating *scleraxis*, *tenomodulin*, and *collagen types I* and *III*, and upregulating *MMP3* levels^33^. Here, injured HH43 tendons upregulated *IL-1β* but not *IL-1R1*, whereas injured HH40 tendons upregulated *IL-1R1* but not *IL-1β* levels, relative to non-injured controls (Fig. 6). Based on these results, HH40 healing might involve more IL-1 signaling and downstream effects than HH43 healing, but this needs further investigation. This is interesting because scarlessly healing sheep fetal tendons exhibit minimal pro-inflammatory response to injury compared to postnatal tendons^7^. Therefore, one might expect injured HH40 tendons to have a reduced pro-inflammatory response compared to older injured HH43 tendons. However, the sheep study focused on only later timepoints^7^, and thus may have missed an early inflammatory response. Interestingly, H&E-staining revealed cells with smaller, rounder, and darker nuclei and pinker cytoplasm (Fig. 2) which are characteristic of leukocytes^7,65–67^, but cell surface marker characterization would more accurately evaluate cell populations. Future studies should also examine IL-1β protein levels and IL-1R1 localization on the cell membrane and perturb IL-1β and IL-1R1 to determine their roles during embryonic tendon healing.

While we discussed various limitations of this study and potential next steps throughout this report, we have additional recommendations. Future studies should perform flow cytometry characterization to identify immune cell populations, followed by cell-depletion studies to determine their contributions to embryonic tendon healing. To determine the regenerative capacity of each embryonic stage, longer healing durations should be evaluated. We discovered expression of phenotype markers and ECM development during healing is embryonic stage-dependent, suggesting earlier and later stage embryonic tendons may heal via different mechanisms. Thus, transcriptomic and proteomic characterizations followed by perturbation experiments should be performed at various timepoints following injury at specific embryonic stages, which could identify embryonic stage-specific pathways and regulators of tendon healing outcomes. Finally, the in vivo model should be more comprehensively characterized in future studies, including longer timepoints and analyses of gene expression, mechanical properties, and other tissue properties.

Taken together, our studies established the feasibility of in vivo and ex vivo chick embryo tendon injury models to study tendon healing at different developmental stages. Embryonic tendons possess a remarkable ability to heal rapidly both in vivo and ex vivo, with healing occurred more rapidly in vivo. Studies with these in vivo and ex vivo chick embryo tendon injury models could enable new insights into accelerated, and potentially regenerative, healing of tendons. We anticipate using these novel models to elucidate novel mechanisms of tendon healing during functional tissue formation to inform the future development of therapeutics to regeneratively heal adult tendons.

## Data availablity

The datasets generated during the current study are available from the corresponding author on reasonable request.
